# Correlates of fruit, vegetable, soft drink, and snack intake among adolescents: the ESSENS study

**DOI:** 10.3402/fnr.v60.32512

**Published:** 2016-09-20

**Authors:** Mekdes K. Gebremariam, Sigrun Henjum, Laura Terragni, Liv Elin Torheim

**Affiliations:** Department of Nursing and Health Promotion, Faculty of Health Sciences, Oslo and Akershus University College of Applied Sciences, Oslo, Norway

**Keywords:** correlates, dietary behaviors, home, school, neighborhood, environment

## Abstract

**Background:**

Identifying modifiable correlates of dietary behaviors is of utmost importance for the promotion of healthy dietary behaviors.

**Objective:**

This study explores individual, home, and school/neighborhood environmental correlates of dietary behaviors (intake of fruits, vegetables, soft drinks, and unhealthy snacks) among adolescents.

**Methods:**

In total, 742 adolescents with a mean age of 13.6 (SD=0.3) were included in this cross-sectional study conducted in 11 secondary schools located in the eastern part of Norway. A web-based questionnaire was used to collect data. Univariable and multivariable linear regression analyses were used to explore factors associated with the dietary behaviors included.

**Results:**

A higher frequency of food/drink purchase in the school canteen was related to a higher consumption of soft drinks and snacks. A higher frequency of food/drink purchase in shops around schools during break or recess was related to a higher consumption of snacks. A higher frequency of food/drink purchase in shops around the neighborhood on the way to and from school was related to a higher consumption of soft drinks. Perceived parental modeling and perceived accessibility at home were found to be positively associated with all dietary behaviors. Perceived parental rules were inversely associated with soft drink and snack consumption; self-efficacy related to healthy eating was positively associated with fruit and vegetable consumption. Other included school and neighborhood environmental correlates were not associated with the dietary behaviors.

**Conclusions:**

There is a need to address the food purchasing behavior of the adolescents using different approaches. The findings also highlight the important role of parents and the home environment for healthy and unhealthy dietary behaviors of adolescents.

One of the major public health challenges globally today is the pandemic of overweight and obesity. The problem is particularly pronounced among youth who can suffer from both short- and long-term complications (1–4) including tracking of body weight into adulthood (5). Unhealthy dietary behaviors are among the multiple interacting forces driving this pandemic (6). In this regard, favorable changes in the consumption of fruits, vegetables, soft drinks, and sweets have been documented in the past decade in Norway (7). However, the consumption of fruits and vegetables remains below what is recommended for health, and the consumption of added sugar is high (7–9). It is therefore of utmost importance to investigate the factors influencing dietary behaviors among adolescents.

Ecological models posit that health behaviors are influenced by factors at multiple levels including individual, interpersonal/social, and environmental levels (10). In this regard, individual factors such as preferences and self-efficacy have been identified as important correlates of dietary behaviors; the home environment has also been found to play a crucial role in determining children and adolescents’ dietary behaviors (11–16). However, wider environmental (e.g. school and neighborhood) correlates of dietary behaviors have been less explored, although the past decade has seen an increase in studies focusing on such correlates (16). A systematic review of reviews documented that no school, neighborhood, or societal factors were consistently related to dietary behaviors among youth (12). Another systematic review concluded that there was little evidence supporting an effect of the retail environment around school on purchasing behavior or on consumption behavior (17). Many studies using multilevel modeling also found low school-level variance in dietary behaviors of children once individual-level characteristics were controlled (18–23). Such findings have at least partly been ascribed to a lack of high-quality studies (17) as well as to the fact that important environmental factors have not been explored. In addition, significant differences exist in the food environment across different settings, in particular in relation to the foods/drinks available at school, contributing to inconsistencies in results. Most studies of school-level correlates of dietary behaviors have been conducted in the USA where the school food environment is different from the European school food environment in general, and the Norwegian school food environment in particular. In Norway, there are no free school lunch programs, and it was documented that 95% of fifth to seventh graders bring their lunch from home (24). It is prohibited to have vending machines in schools; foods/drinks are offered via canteens/food booths that might have variable services and opening hours. Therefore, results regarding the influence of the school food environment from countries such as the USA cannot be directly extrapolated to the Norwegian setting. In addition, a recent umbrella review of correlates of dietary behaviors among youth concluded that groups of correlates have often been studied in isolation (16). There is therefore a need for studies that explore simultaneously multiple levels of influence.

Against this background, this study aimed to explore individual, home environmental, and school/neighborhood correlates of the consumption of fruits, vegetables, soft drinks, and unhealthy snacks among adolescents. It was hypothesized, based on existing literature, that the individual and home environmental correlates included would have the most significant contribution in explaining the dietary behaviors of the adolescents. It was also hypothesized that school and neighborhood environmental variables would independently contribute to the prediction of the dietary behaviors included. The participants in this study are young adolescents who are gradually gaining autonomy and are therefore likely to be increasingly influenced by food environment other than the home food environment.

## Methods

### Design and sample

The participants in this study were pupils from 11 secondary schools participating in the Environmental determinantS of dietary BehaviorS among adolescENtS (ESSENS) cross-sectional study. All 12 secondary schools in the Øvre Romerike region located in the eastern part of Norway were invited to participate in the study, and 11 accepted the invitation. In total, 1,163 adolescents in the eighth grade were invited to participate in this study and a total of 781 (67%) received parental consent for participation. In total, 742 adolescents (64% of those invited and 95% of those with parental consent) participated in the study.

Ethical clearance for the study was obtained from the Norwegian Social Science Data Service. Written informed consent was obtained from all parents of participating students.

### Data collection and measures

A web-based questionnaire was used to collect data from the adolescents. The questionnaires were filled in at school and took approximately 30 to 45 min to complete. Research group members were present during data collection to answer questions and make sure adolescents responded independently from each other. The questionnaire was pretested among a group of adolescents (*n*=23) of the same age as the study participants, prior to the main study.

## Measures

### Dietary behaviors

Intake of carbonated sugar-sweetened soft drinks (hereafter referred to as soft drinks) during weekdays was assessed using a frequency question with categories ranging from never/seldom to every weekday, and amount in glasses (from one to four or more). Weekend intake was assessed using a question about the amount of soft drink consumed with categories ranging from never/seldom to seven glasses or more. As soft drinks are usually sold in half liter bottles or cans, the participants were informed that ½ liter equals three glasses. Weekday and weekend intakes were summed up to create a weekly intake variable. The questions assessing the intake of soft drinks have been validated among 9- and 13-year-old Norwegians using a 4-day precoded food diary as the reference method, and moderate Spearman's correlation coefficients were obtained (25). However, separate assessment of weekday and weekend intake was not made in the validation study.

Consumption of fruits and vegetables (raw and cooked) was assessed using frequency questions with eight response categories ranging from never/seldom to three times per day or more. The questions assessing intake of fruits and vegetables were validated among 11-year-olds with a 7-day food record as the reference method and were found to have a satisfactory ability to rank subjects according to their intake of fruits and vegetables (26).

The consumption of snacks [sweet snacks (candies/chocolate), fatty snacks (e.g. potato chips), and sweet biscuits/muffins and similar] was assessed using three questions with seven response categories ranging from never/seldom to two times per day or more; frequencies of consumption of all the snacks were added to create a total snack intake variable.

Acceptable to moderate test–retest reliability was obtained for these measures of dietary behaviors in a previous Norwegian study conducted among 11-year-olds (27).

Perceived accessibility of vegetables was assessed using a scale with four items (e.g. ‘we vary the types of vegetables served for dinner during a week’); perceived accessibility of fruits was assessed using a scale with three items (e.g. ‘we vary the types of fruits that we have during the course of the week’); perceived accessibility of soft drinks was assessed using a scale with three items (e.g. ‘we usually have soft drinks for dinner on weekend days’); and perceived accessibility of snacks was assessed using a scale with three items (e.g. ‘we usually have sweet or fatty foods served for dessert or as snacks on weekdays’). A five-point Likert scale with answer categories ranging from ‘totally disagree to totally agree’ was used.

Perceived parental rules related to the consumption of vegetables were assessed using a scale with two items (e.g. ‘I can eat vegetables whenever I want’); a similar scale was used to assess the perceived parental rule related to fruit consumption. Perceived parental rules for the consumption of soft drinks were assessed using a scale with four items (e.g. ‘we have rules for when I can drink soft drinks’); perceived parental rules related to snack consumption was assessed using a single item (‘when we have sweet or fatty snacks available, I can eat as much as I want’). A five-point Likert scale with answer categories ranging from ‘totally disagree to totally agree’ was used.

The items assessing perceived rules and accessibility of vegetables and soft drinks have previously shown evidence of validity among 14-year-olds (28).

Perceived parental modeling of fruit intake and of vegetable intake was assessed using a scale with two items. The measures were adopted from the Pro Children study and were found to have adequate test–retest reliability among 10–11-year-old European children (29). Parental modeling of soft drink consumption was assessed using the question ‘How often do your parents drink soft drinks with sugar?’ This question has shown evidence of test–retest reliability among 10–12-year-old European children (30). A similar scale was used to assess parental consumption of unhealthy snacks.

Self-efficacy related to the consumption of healthy foods was assessed using a scale with six items [e.g. Whenever I have a choice of the food I eat …, I find it difficult to choose low-fat foods (e.g. fruit or skimmed milk rather than ‘full cream milk’)]. The scale has been found to have adequate reliability and factorial validity among 13-year-olds (31).

All scales had acceptable to moderate internal consistency reliability in the present sample (ICC: 0.50–0.80).

The adolescents were asked about the presence of food sales outlets (e.g. supermarket, kiosk, and gas station) in a walking distance from their school (with answer categories ‘none’, ‘yes, one’, ‘yes, two’, and ‘yes, more than two’). The question was adopted from a previous Norwegian study (23). They were asked how often they bought foods/drinks in these shops during breaks or recess. They were also asked how often they purchased foods or drinks from school canteens and on their way to and from school (answer categories ranging from ‘never’ to ‘every day’). The frequency of purchase of foods/drinks was then recategorized into never, once a week, and more than once a week based on the distribution of the variables.

The perceived availability of fruits, vegetables, and unhealthy snacks in shops around school and in shops in the neighborhood was also assessed (e.g. a lot of fresh and varied fruits and vegetables that I like are available). Perceived accessibility of fruits and vegetables in these shops was asked using the question ‘There are a lot of fruits and vegetables in a form that is easy to consume’. Perceived accessibility of soft drinks or snacks compared to fruits or vegetables in shops around schools or in the neighborhood was assessed using the question ‘It is easier to find soft drinks and snacks in the shops than it is to find fruits or vegetables’. Perceived price of soft drinks and snacks compared to fruits and vegetables was explored using the question ‘It is cheaper to buy soft drinks or snacks (e.g. biscuits, chips) than it is to buy fruits or vegetables’. Answer categories ranged from ‘completely agree’ to ‘completely disagree’.

Information at school regarding food/nutrition was assessed using a scale with two items (e.g. ‘food and nutrition is a topic that my teachers talk about often’). The internal consistency for the scale was moderate (ICC=0.78). Perceived rules regarding foods and drinks allowed at school were assessed using the question: ‘We have rules at school regarding the foods/drinks we should or should not bring to school’ (answer categories ranging from ‘completely agree’ to ‘completely disagree’). The question was adopted from a previous Norwegian study (23).

### Sociodemographic correlates

Information on parental education was gathered as part of the parental informed consent for the adolescent. It was categorized into low (12 years of education or less, which corresponded to secondary education or lower) and high (13 years of education and more, which corresponded to university or college attendance). Educational status of the parent with the longest education or else the one available was used in the analyses.

Participants were divided into either ethnic Norwegian or ethnic minority. Ethnic minorities were defined as those having both parents born in a country other than Norway (32).

### Statistical analyses

Because schools were the unit of measurement in this study, we checked for clustering effect through the linear mixed model procedure. Only ≤3% of the unexplained variance in the dietary behaviors investigated was at the school level. Hence, adjustment for clustering effect was not done.

Descriptive analyses were first conducted. Univariable linear regression analyses were then used to explore factors at the individual (including sociodemographic characteristics), interpersonal, home environmental, and school/neighborhood environmental levels associated with the different dietary behaviors. Thereafter, variables significant at the 0.2 level on univariable regression were entered in multivariable regression models. Assumptions for the analyses were checked and considered acceptable. All analyses were conducted using SPSS version 22.

## Results

The sociodemographic characteristics of the adolescents are described in Table 1. The mean age was 13.6 (SD=0.3), and 53% of participants were females. Only 9% of adolescents were of an ethnic minority background and 40% had parents with a low level of education. The mean consumption of soft drinks was 700 mL/week. The frequency of fruit, vegetable, and snack consumption was 6.9, 8.8, and 4.5 times/week, respectively.

**Table 1:** Sociodemographic characteristics and dietary behaviors of the study sample (*n*=742)

Age (years)	13.6 (0.3)
Gender (% girls)	53
Parental education (% low^a^)	40
Ethnicity (% ethnic minority)	9
Soft drink consumption (dl/week)	7.0 (6.5–7.4)
Fruit intake (times/week)	6.9 (6.5–7.4)
Vegetable intake (times/week)	8.8 (8.2–9.2)
Snack intake (times/week)	4.5 (4.2–4.7)

Results are presented as mean and confidence interval or percentages.

a Low education is defined as having 12 years of education or less.

Table 2 shows the results of the descriptive analyses of the correlates included in the study. The lowest mean values were obtained for the perceived accessibility of unhealthy snacks and soft drinks at home. The highest values were obtained for permissive parental rules related to vegetable and fruit intake. The mean number of food sales outlets around schools was 2.2, and only 4% of participants reported that there were no food sales outlets in a walking distance from their school. In total, 73% of participants reported that they never purchased foods/drinks in shops around schools during breaks or recess; 11% reported doing so twice or more per week. Similarly, 66% of participants reported that they never purchased foods/drinks from neighborhood shops on their way to and from school, whereas 24% and 10% reported doing so once a week and twice or more per week, respectively. No food/drink purchase in school canteen was reported by 67% of participants; 20% reported purchasing foods/drinks once a week and 13% twice or more per week.

**Table 2:** Descriptive analysis of potential correlates of dietary behaviors among study participants (*n*=742)

	Mean (SD) or %	Range
Perceived parental modeling of soft drink intake	2.71 (0.83)	1–5
Perceived accessibility of soft drinks at home	2.57 (1.14)	0–5
Perceived parental rules related to soft drinks	4.28 (1.11)	1–6
Perceived parental modeling of snack intake	2.70 (0.62)	1–5
Perceived accessibility of snacks at home	2.49 (0.79)	1–5
Perceived parental rules related to snacks	3.90 (1.23)	1–5
Perceived parental modeling of vegetable intake	3.68 (0.97)	1–5
Perceived accessibility of vegetables at home	4.09 (0.77)	1–5
Perceived parental rules related to vegetables	4.55 (0.71)	1–5
Perceived parental modeling of fruit intake	4.14 (0.87)	1–5
Perceived accessibility of fruits at home	3.89 (0.78)	1–5
Perceived parental rules related to fruits	4.56 (0.68)	1–5
Self-efficacy related to healthy eating	3.48 (0.65)	1–5
Number of food sales outlets around school^a^	2.19 (0.93)	0–3
Food/drink purchase in school canteen		
Never	67%	
Once a week	20%	
Twice or more per week	13%	
Food/drink purchase in neighborhood shops^b^		
Never	66%	
Once a week	24%	
Twice or more per week	10%	
Food/drink purchase in shops around school^c^		
Never	73%	
Once a week	16%	
Twice or more per week	11%	
Perceived FV availability in neighborhood shops	4.24 (1.02)	1–5
Perceived FV accessibility in neighborhood shops	4.09 (1.08)	1–5
Perceived accessibility of SD and snacks in neighborhood	3.03 (1.24)	1–5
Perceived availability of SD and snacks in neighborhood	3.88 (1.19)	1–5
Perceived price of SD and snacks compared to FV	3.08 (1.23)	1–5
Perceived school food rules	3.56 (1.36)	1–5
Information at school regarding food and nutrition	2.68 (1.05)	1–5

SD, soft drinks; FV, fruits and vegetables.

a In a walking distance from school

b on the way to and from school

c during breaks or recess.

In the final adjusted models, the following factors were found to be associated with soft drink consumption (dl/week): gender (reference=female) B=1.33 (confidence interval (CI): 0.48–2.17), perceived parental modeling B=0.63 (CI: 0.06–1.21), perceived accessibility at home B=1.47 (CI: 1.02–1.92), perceived parental rules B=−1.58 (CI: −2.01 to −1.15), purchase of food/drink at the school canteen (high vs. low) B=2.10 (CI: 0.74–3.45), and purchase of food/drinks on the way to and from school (high vs. low) B=2.87 (CI: 1.31–4.43). The factors found to be associated with snack consumption in the final adjusted models were perceived parental modeling B=0.43 (95% CI: 0.01–0.85), perceived accessibility at home B=1.0 (95% CI: 0.66–1.35), perceived parental rules B=−0.22 (95% CI: −0.43 to −0.01), self-efficacy related to healthy eating B=−0.90 (95% CI: −1.29 to −0.51), purchase of food/drink in the school canteen (high vs. low) B=0.92 (95% CI: 0.13–1.71), and food/drink purchase around school during recessand breaks (high vs. low) B=1.57 (95% CI: 0.73–2.41). Table 3 shows the results of the multivariable regression analyses exploring correlates of soft drink and snack consumption.

**Table 3:** Correlates of soft drink and snack consumption among adolescents, *n*=742

	B and CI	*p*
Soft drink consumption (dl/week)		
Gender (males)	1.33 (0.48–2.17)	0.002
Parental education	−0.21 (−1.08–0.66)	0.64
Perceived parental modeling	0.63 (0.06–1.21)	0.03
Perceived accessibility at home	1.47 (1.02–1.92)	<0.001
Perceived parental rules	−1.58 (−2.01 to −1.15)	<0.001
Self-efficacy regarding healthy eating	−0.58 (−1.24–0.08)	0.08
Perceived FV availability in neighborhood shops	−0.40 (−0.80–0.02)	0.06
Food/drink purchase in school canteen (high)	2.10 (0.74–3.45)	0.002
Food/drink purchase in school canteen (medium)	0.03 (−1.06–1.12)	0.96
Food/drink purchase in neighborhood shops^a^ (high)	2.87 (1.31–4.43)	<0.001
Food/drink purchase in neighborhood shops^a^ (medium)	−0.41 (−1.44–0.62)	0.44
Food/drink purchase in shops around school^b^ (high)	−0.92 (−2.36–0.52)	0.21
Food/drink purchase in shops around school^b^ (medium)	−0.85 (−2.0–0.30)	0.15
Intake of snacks (times/week)		
Perceived parental modeling	0.43 (0.01–0.85)	0.046
Perceived accessibility at home	1.0 (0.66–1.35)	<0.001
Perceived parental rules	−0.22 (−0.43 to −0.01)	0.04
Self-efficacy regarding healthy eating	−0.90 (−1.29 to −0.51)	<0.001
Perceived availability in neighborhood shops	0.14 (−0.08–0.35)	0.21
Perceived price compared to fruits and vegetables	0.20 (0.00–0.40)	0.05
Food/drink purchase in school canteen (high)	0.92 (0.13–1.71)	0.02
Food/drink purchase in school canteen (medium)	−0.27 (−0.9–0.37)	0.42
Food/drink purchase in neighborhood shops^a^ (high)	0.29 (−0.64–1.21)	0.54
Food/drink purchase in neighborhood shops^a^ (medium)	−0.28 (−0.89–0.32)	0.36
Food/drink purchase in shops around school^b^ (high)	1.57 (0.73–2.41)	<0.001
Food/drink purchase in shops around school^b^ (medium)	0.15 (−0.53–0.83)	0.67

FV, fruits and vegetables.

a On the way to and from school

b during recess or breaks.

Results were obtained using multiple linear regression analyses, and only factors significant at the 0.2 level on univariable regression were included in this model. Frequency of food purchase is defined as high when it occurs twice or more per week and as medium when it occurs once a week. The reference category includes those who report no purchase of food/drinks.

The factors found to be associated with fruit intake in the final adjusted models were perceived parental modeling B=0.54 (95% CI: 0.02–1.06), perceived accessibility at home B=1.38 (95% CI: 0.76–1.99), and self-efficacy regarding healthy eating B=2.69 (95% CI: 2.01–3.38). The factors found to be associated with vegetable intake were perceived parental modeling B=0.58 (95% CI: 0.09–1.07), perceived accessibility at home B=2.98 (95% CI: 2.28–3.68), and self-efficacy regarding healthy eating B=2.20 (95% CI: 1.46–2.94). Table 4 shows the results of the multivariable regression analyses exploring correlates of fruit and vegetable consumption.

**Table 4:** Correlates of fruit and vegetable consumption among adolescents, *n*=741

	B and CI	*p*
Fruit consumption (times/week)		
Ethnicity	1.28 (−0.23–2.79)	0.09
Perceived parental modeling	0.54 (0.02–1.06)	0.04
Perceived accessibility at home	1.38 (0.76–1.99)	<0.001
Perceived parental rules	−0.26 (−0.93–0.41)	0.45
Self-efficacy regarding healthy eating	2.69 (2.01–3.38)	<0.001
Perceived FV availability in neighborhood shops	0.07 (−0.36–0.50)	0.74
Perceived food rules at school	−0.23 (−0.54–0.08)	0.15
Vegetable consumption (times/week)		
Perceived parental modeling	0.58 (0.09–1.07)	0.02
Perceived accessibility at home	2.98 (2.29–3.68)	<0.001
Perceived parental rules	0.08 (−0.61–0.77)	0.82
Self-efficacy regarding healthy eating	2.20 (1.46–2.94)	<0.001
Perceived FV availability in neighborhood shops	−0.32 (−0.77–0.12)	0.15

FV, fruits and vegetables.

Results were obtained using multiple linear regression analyses, and only factors significant at the 0.2 level on univariable regression were included in this model.

## Discussion

The aim of this study was to explore individual, home environmental, and school/neighborhood environmental correlates of fruit, vegetable, soft drink, and unhealthy snack consumption among adolescents. The adolescents’ food purchasing behavior at school and in the neighborhood was associated with soft drink and snack consumption. The other school and neighborhood environmental correlates were not associated with the dietary behaviors of the adolescents. Parental modeling and perceived accessibility at home were found to be positively associated with all the dietary behaviors. Parental rules were associated with soft drink and snack consumption; self-efficacy related to healthy eating was associated with fruit and vegetable consumption.

As hypothesized, home environmental variables were found to be the most important correlates of the dietary behaviors included. As previously documented in the literature, parental modeling (11–14) and perceived accessibility at home (13, 15) were found to be positively associated with all the dietary behaviors included. Self-efficacy related to healthy eating was also strongly positively related to the consumption of fruits and vegetables and inversely related to the consumption of unhealthy snacks. Self-efficacy has been found to be a strong correlate of healthy behaviors among adolescents including dietary behaviors (15). Parents can play a role in the enhancement of their adolescents’ self-efficacy toward healthy eating that would result in favorable dietary behaviors, which might further enhance self-efficacy. There were also parental educational differences in soft drink consumption on univariable analysis. This association became insignificant in the final adjusted model, indicating that variables included in the model were potentially mediators of the socioeconomic differences in soft drink consumption. Indeed, separate analyses (results not shown) showed that there were socioeconomic differences in the correlates that were significantly associated with soft drink consumption. Factors such as parental modeling and accessibility at home have previously been found to mediate socioeconomic differences in soft drink consumption (33–35).

The frequency of food purchase in the school canteen was positively related to the intake of unhealthy snacks and soft drinks. The Norwegian recommendations for school food state that the offer of cakes and other sweet or fatty products be limited to special occasions. These recommendations also state that soft drinks should not be offered at school (36). However, in a qualitative component of the ESSENS study, which included observations of canteens in selected schools, it was found that some unhealthy foods and drinks were offered in some schools, in addition to healthier food alternatives (unpublished data). A previous study from the USA found that middle school students purchased unhealthy foods such as chips, soda, and soft drinks at school even when healthier alternatives were available (37). In another study, availability at school was related to the consumption of soft drinks and unhealthy snacks, but not with fruit and vegetable consumption (38). Limiting the availability of unhealthy foods and drinks from school canteens by ensuring that national guidelines for foods at school are properly implemented is therefore crucial.

The frequency of food purchase in neighborhood shops on the way to and from school was positively related to the consumption of soft drinks. The frequency of food purchase in shops around schools during breaks or recess was positively related to the consumption of unhealthy snacks. It is worth noting that there might be some overlap between these shops, as the shops around school might be the ones most accessible on the way to and from school for some, in particular those living in close proximity of their school. Food purchase away from home has been associated with unfavorable eating behaviors and weight outcomes in previous studies, although the type of food purchased and of food outlets explored varied between studies (39–41). In this regard, parents should encourage their children to purchase healthy foods and whenever possible limit their adolescents’ food purchase by providing fewer financial resources for doing so. There was also a borderline statistically significant inverse association between the perceived price of snacks compared to fruits and vegetables and the consumption of snacks, that is, those who perceived the price of snacks to be lower consumed more snacks. Food prices have been shown to be key correlates of healthy and unhealthy food consumption (42–44) and can therefore serve as one of the entry points for interventions aimed at improving adolescents’ dietary behaviors.

In this study, school and neighborhood environmental variables did not appear to have a significant influence on the dietary behaviors of the adolescents, in particular fruit and vegetable consumption. There might be several reasons for these findings. Existing data indicate that 95% of fifth to seventh graders in Norway bring their school lunch from home (24) which is likely similar among eighth graders. Therefore, most of the consumption of fruits and vegetables is likely to be either at home or from fruits and vegetables brought from home. It is also possible that there is homogeneity in the factors of interest between schools and neighborhoods although there was diversity in the perceptions reported. Consequently, actual school and neighborhood correlates might not have had enough variability that would allow for associations with dietary behaviors to be picked, for example, shops around schools might have similar availability/accessibility of foods across the included schools. Indeed, some ceiling effect was apparent for the measure of perceived fruit and vegetable availability in neighborhood shops. Future studies combining objective and subjective measures of the school and neighborhood environment in the same sample are needed in this regard. Such studies would allow for the assessment of similarities and differences in associations when subjective and objective measures are used. The latter might provide a more accurate assessment; however, how these environmental factors are perceived by the adolescents might be more relevant in influencing their behaviors.

The strengths of the study include the large number of correlates at multiple levels that were included. The sample size was also large; the response rate at the school level was very high and was moderate at the parental level. The weaknesses include the cross-sectional data that does not allow for any causal inference to be made. The use of self-report can lead to problems of validity and reliability, in particular among young children. Several of the correlates included, however, had displayed evidence of reliability and/or validity in previous studies among adolescents in a similar age group. There is, however, a scarcity of validity and reliability tested measures assessing the perceived school and neighborhood food environment including the measures used in this study. Finally, only carbonated sugar-sweetened soft drinks were included in this study; future studies should include other sugar-sweetened beverages such as cordials and energy drinks.

## Conclusions

Food purchasing behavior at school and on the way to and from school was found to be related to the intake of soft drinks and snacks. Perceived accessibility and parental modeling were related to all dietary behaviors; parental rules were related to soft drink and snack intake. Other school and neighborhood environmental variables were unrelated to the dietary behaviors included. There is therefore a need to address the food purchasing behavior of the adolescents using different approaches such as limiting the availability of unhealthy foods and drinks from school canteens. The findings also highlight the important role of parents and the home environment for healthy and unhealthy dietary behaviors of adolescents.
